# Novel Fabry-Pérot Filter Structures for High-Performance Multispectral Imaging with a Broadband from the Visible to the Near-Infrared

**DOI:** 10.3390/s25196123

**Published:** 2025-10-03

**Authors:** Bo Gao, Tianxin Wang, Lu Chen, Shuai Wang, Chenxi Li, Fajun Xiao, Yanyan Liu, Weixing Yu

**Affiliations:** 1 Key Laboratory of Spectral Imaging Technology of Chinese Academy of Sciences, Xi’an Institute of Optics and Precision Mechanics, Xi’an 710119, China; gaobo_101@opt.ac.cn (B.G.); chenlu2022@opt.ac.cn (L.C.); wangshuai1@opt.ac.cn (S.W.); lichenxi@opt.ac.cn (C.L.); 2 Center of Materials Science and Optoelectronics Engineering, University of Chinese Academy of Sciences, Beijing 100049, China; 3 Key Laboratory of Light Field Manipulation and Information Acquisition, Ministry of Industry and Information Technology, and Shaanxi Key Laboratory of Optical Information Technology, School of Physical Science and Technology, Northwestern Polytechnical University, Xi’an 710129, China; fjxiao@nwpu.edu.cn; 4 National Key Laboratory of Electromagnetic Space Security, Tianjin 300308, China; lyy13@163.com

**Keywords:** snapshot multispectral imaging, multispectral filter arrays, broadband

## Abstract

The integration of a pixelated Fabry–Pérot filter array onto the image sensor enables on-chip snapshot multispectral imaging, significantly reducing the size and weight of conventional spectral imaging equipment. However, a traditional Fabry–Pérot cavity, based on metallic or dielectric layers, exhibits a narrow bandwidth, which restricts their utility in broader applications. In this work, we propose novel Fabry–Pérot filter structures that employ dielectric thin films for phase modulation, enabling single-peak filtering across a broad operational wavelength range from 400 nm to 1100 nm. The proposed structures are easy to fabricate and compatible with complementary metal-oxide-semiconductor (CMOS) image sensors. Moreover, the structures show low sensitivity to oblique incident angles of up to 30° with minimal wavelength shifts. This advanced Fabry–Pérot filter design provides a promising pathway for expanding the operational wavelength of snapshot spectral imaging systems, thereby potentially extending their application across numerous related fields.

## 1. Introduction

Multispectral imaging systems capture spectral information across various wavelengths, offering detailed insights into scene features that are not visible to traditional RGB cameras [1,2]. The capability could be used for more precise recognition and analysis of targets, making multispectral imaging valuable in fields such as remote sensing, material analysis, and camouflage detection [3]. However, conventional multispectral imaging systems based on prisms or gratings for light dispersion face limitations due to bulky optical systems and slow imaging speeds, which are caused by the need for mechanical movement during data acquisition. The development of CMOS devices has revolutionized multispectral imaging by enabling the integration of multispectral filter arrays (MSFAs) with CMOS sensors [4,5]. The single filter array could be used to cover multiple spectral bands within a compact footprint, with each pixel on the CMOS sensor capturing data from a distinct spectral channel [6]. This setup enables snapshot multispectral data acquisition. The performance of the MSFAs is crucial, as it directly affects the overall capabilities of systems [7,8]. The primary challenge is designing and fabricating miniature filter structures on CMOS sensors, typically the pixel size of which is only a few micrometers, while still meeting the required detection specifications [9,10].

Recent advancements in nanofabrication have made it possible to create miniature MSFAs with individual filter sizes smaller than 10 μm using thin film stacks or metal-dielectric metasurfaces [11]. However, designing these MSFAs involves balancing several factors, including transmittance, spectral resolution, color purity, operational bandwidth, and angular response, all of which impact manufacturability [7]. Various resonance mechanisms are employed in miniature MSFAs to filter incoming light, such as plasmonic resonance, Mie resonance, guided-mode resonance, and cavity resonance [12]. Plasmonic resonance-based MSFAs, which typically use metallic metasurfaces, offer a broad operational wavelength range and are relatively straightforward to manufacture [13]. Nevertheless, their transmission efficiency is often limited by metal absorption, which can hinder the color purity and narrow linewidth, requiring more complex spectral reconstruction algorithms [14,15]. Guided-mode resonance filters, which combine diffraction gratings with waveguide resonance, can achieve a narrow bandwidth and high purity but are highly sensitive to polarization and incident angle [7,16,17,18].

One of the most prevalent designs for MSFAs is based on Fabry–Pérot cavity resonators [7]. These resonators are optical cavities sandwiched between two distributed Bragg reflectors (DBRs) or metal mirrors. In contrast to diffraction-based filters, Fabry–Pérot resonators are relatively insensitive to polarization and incident angle [19]. They are further characterized by their excellent spectral resolution and monochromaticity. However, their operational bandwidth is limited by the characteristics of the reflectors. High-reflectivity DBRs exhibit a narrow reflection bandwidth, typically around 200 nm in the visible range [20,21]. In contrast, metal mirrors exhibit substantial material absorption, resulting in markedly lower transmittance at certain wavelengths [22]. But, by precisely adjusting the thickness of each thin film through optimized design algorithms, the transmission efficiency of the filter structure can be significantly enhanced [23,24]. Additionally, the resonance mechanism inside the cavity can produce multiple-order resonances, which constrain the free spectral range (FSR), consequently limiting the operational bandwidth [25,26]. Suppressing certain resonance peaks through structural design or precise film control to broaden the FSR is an effective solution, but the extent of FSR broadening remains limited [12,27].

In this study, we propose a novel dielectric–metal mirror that maintains stable transmittance and reflectance across a broad wavelength range of 400–1100 nm. Based on this mirror, we construct a Fabry–Pérot resonator that achieves excellent transmittance exceeding 50% and a full width at half maximum (FWHM) of less than 25 nm within the same spectral range. Furthermore, we increase the FSR by incorporating a high-refractive-index thin film inside the cavity, which subsequently enhances its overall performance. This film introduces phase modulation and optical interference control; a high-refractive-index thin film inside the cavity is used to increase the separation between resonant frequencies rather than to eliminate odd-order resonances as in [12]. Consequently, the FSRs of the filter structures are extended to 700 nm, enabling single-peak filtering across the broad 400–1100 nm range. We focused solely on adjusting the resonator cavity thicknesses and adding an internal film layer. This design ensures all films in our structures can be deposited without etching during fabrication, significantly reducing manufacturing complexity. The performance of the proposed filter structures has been validated through deposition techniques, demonstrating their feasibility. Additionally, we apply a basic color clustering algorithm to illustrate the advantages of this ultra-broadband imaging device in classification and recognition tasks, underscoring its potential for imaging applications.

## 2. Design and Principle

To achieve a multispectral filter array with wide operational bandwidth, narrow linewidth, high transmittance, and excellent color purity on a single detector, we have developed an enhanced Fabry–Pérot filter structure, in which high-refractive-index thin films on both the metallic mirrors and within the resonant cavity are incorporated.

### 2.1. Construction of the Resonant Cavity

MDR is the most critical factor in determining the performance of Fabry–Pérot filter structures, as its parameters directly influence the FWHM of the transmission peak and overall transmittance of the device. The following formulas are for calculating the FWHM and the resonance wavelength *λ_m_* [28]:

(1) $$\mathrm{FWHM}=\frac{2nh(1-R)}{m^{2}\pi \sqrt{R}}$$

(2) $$\lambda_{m}=\frac{2nh\cos\theta }{m+\varphi /2\pi }$$

In these equations, *n* represents the refractive index of the material inside the cavity, *h* is the cavity thickness, *R* is the mirror reflectivity, *m* represents the order of resonance, *θ* is the refraction angle, and *φ* is the cumulative reflection phase from the two reflectors.

According to Equations (1) and (2), higher mirror reflectivity results in a narrower FWHM, hence improving the spectral resolution. If absorption remains unchanged, an increase in transmittance leads to a higher peak transmission. Bragg mirrors are multilayer structures that are based on constructive optical interference between alternating material layers to achieve high reflectivity within a specific wavelength band. The greater the refractive index contrast between layers, the broader the reflection bandwidth. However, even with the highest refractive index contrast available, such as between SiO_2_ and TiO_2_, the reflection bandwidth is inherently limited to about 150 nm in the visible and near-infrared spectrum [29]. This restricts the operational bandwidth of filters based on these mirrors to roughly 150 nm. To achieve a broader working bandwidth would require combining multiple designs with varying thicknesses, significantly increasing the manufacturing complexity and cost. Metallic thin-film mirrors, such as those made of silver (Ag), are often used due to their high reflectivity and wide reflection bandwidth [30]. However, metals have higher absorption, especially in the long-wavelength region. This results in reduced overall transmittance, a limitation that becomes increasingly more severe in the near-infrared range [31].

To mitigate the decline in transmittance caused by the absorption of metal films, Peter H. Berning and colleagues introduced the concept of induced transmission [32]. By adding a multilayer dielectric stack behind the metal film, reflection is counteracted, thereby enhancing transmittance at specific wavelengths. Jingyuan Zhu and others also have applied a similar principle to reduce the FWHM of Fabry–Pérot resonators [33]. Based on the design of metal and Bragg mirrors, we have developed a novel MDR to construct the Fabry–Pérot cavity, which combines the advantages of both. In this work, MDR consists of a Ag film in conjunction with a high-refractive-index dielectric layer. The high inherent reflectivity of the metal film is enhanced by the interference effects introduced by the dielectric layer, forming a quasi-Bragg mirror configuration. By simultaneously increasing transmittance and reducing overall reflectance, this design achieves a more stable and broader operational bandwidth, thereby making it well-suited for Fabry–Pérot resonator applications. For thin-film systems, given the thin-film thickness *d*, the characteristic matrix for the film system of Ag (index = *n*_Ag_ − *ik*_ag_) and dielectric (index = *n*_n_) layers can be expressed as follows [28]:

(3) $$M=\left[\begin{array}{cc} \cos\frac{2\pi d_{n}n}{\lambda } & i\frac{\sin\frac{2\pi d_{n}n}{\lambda }}{\left(n_{Ag}-ik_{Ag}\right)}\\ in\sin\frac{2\pi d_{n}n}{\lambda } & \cos\frac{2\pi d_{n}n}{\lambda } \end{array}\right]\left[\begin{array}{cc} \cos\frac{2\pi d_{Ag}(n_{Ag}-ik_{Ag})}{\lambda } & i\frac{\sin\frac{2\pi d_{Ag}(n_{Ag}-ik_{Ag})}{\lambda }}{\left(n_{Ag}-ik_{Ag}\right)}\\ i(n_{Ag}-ik_{Ag})\sin\frac{2\pi d_{Ag}(n_{Ag}-ik_{Ag})}{\lambda } & \cos\frac{2\pi d_{Ag}(n_{Ag}-ik_{Ag})}{\lambda } \end{array}\right]\left[\begin{array}{c} 1\\ \eta_{0} \end{array}\right]=\left[\begin{array}{c} B\\ C \end{array}\right]$$

The reflectance and transmittance of MDR can be calculated using the values of *B* and *C* obtained from Equation (3). The calculation formulas are as follows (*η*_0_ is the refractive index of the medium in the film system environment):

(4) $$R=\left(\frac{\eta_{0}B-C}{\eta_{0}B+C}\right){\left(\frac{\eta_{0}B-C}{\eta_{0}B+C}\right)}^{*}$$

(5) $$T=\frac{4{\eta_{0}}^{2}}{(\eta_{0}B+C){\left(\eta_{0}B+C\right)}^{*}}$$

**Figure 1:**
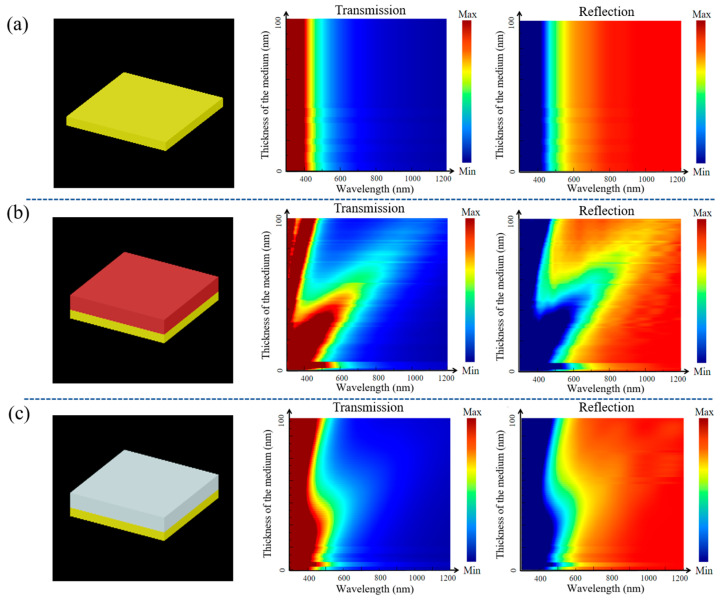
Transmission and reflection of (**a**) Ag mirrors and with (**b**) TiO_2_ and (**c**) SiO_2_ films.

We utilized Ag as the base metal reflector due to its excellent performance across the visible to near-infrared regions. A 40 nm Ag film was paired with dielectric layers made of SiO_2_ and TiO_2_. To assess the impact of these dielectric materials and their thickness on the reflectance and transmittance of MDR, we performed simulations using Ansys Lumerical FDTD 2020 software [34]. The results reveal that introducing dielectric films significantly modifies the optical properties of the MDR, as shown in Figure 1. The transmittance is improved for wavelengths above 500 nm in particular; according to Equations (1) and (3), this enhancement makes the configuration particularly well-suited for Fabry–Pérot resonators. The choice of dielectric material is crucial, as demonstrated in Figure 1. According to Equation (1), when the product of the refractive index (*n*) and thickness (*d*) is held constant, the refractive index becomes the primary variable factor influencing the characteristic matrix of the dielectric layer. As shown in Figure 1, a higher refractive index leads to a greater increase in transmittance. Based on these findings, we selected TiO_2_ as the dielectric material, ensuring stable transmission characteristics across the target spectral range. Moreover, the TiO_2_ film could prevent oxidation of the Ag layer, enhancing the environmental stability of the structure.

**Figure 2:**
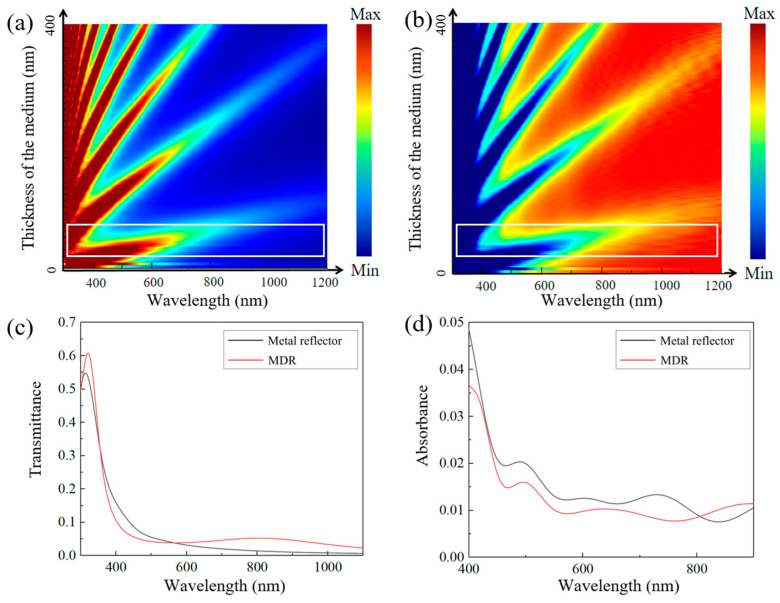
(**a**) Transmittance and (**b**) reflectance of MDR composed of TiO_2_ films of different thicknesses. Difference in (**c**) transmittance and (**d**) absorbance between Ag reflector and MDR.

To determine the optimal TiO_2_ thickness, we conducted a parametric scan ranging from 0 to 400 nm, evaluating its impact on transmittance and reflectance. As shown in Figure 2, increasing the thickness of the dielectric layer introduces distinct transmission enhancement bands. These bands become more pronounced, both in number and slope, as the TiO_2_ layer thickens. However, excessive thickness introduces multiple transmission enhancement bands within the 400–1100 nm range, leading to significant fluctuations in the transmission peaks of the Fabry–Pérot resonator across this spectral region. In contrast, decreasing the thickness makes the bandwidth too broad. To achieve a more stable and continuous transmission response and a narrower bandwidth, the optimal dielectric layer thickness is set between 50 nm and 80 nm, as highlighted by the white box in Figure 2a,b. Figure 2c,d show the reflectance and absorbance of an MDR composed of 76 nm TiO_2_ and 40 nm Ag. Compared to a standalone 40 nm Ag film, the addition of the TiO_2_ layer significantly enhances transmittance across the 500–1100 nm range, producing a smoother and more stable transmittance curve. Furthermore, simulation results reveal a marked reduction in absorption within the 400–800 nm range, further optimizing the overall performance.

**Figure 3:**
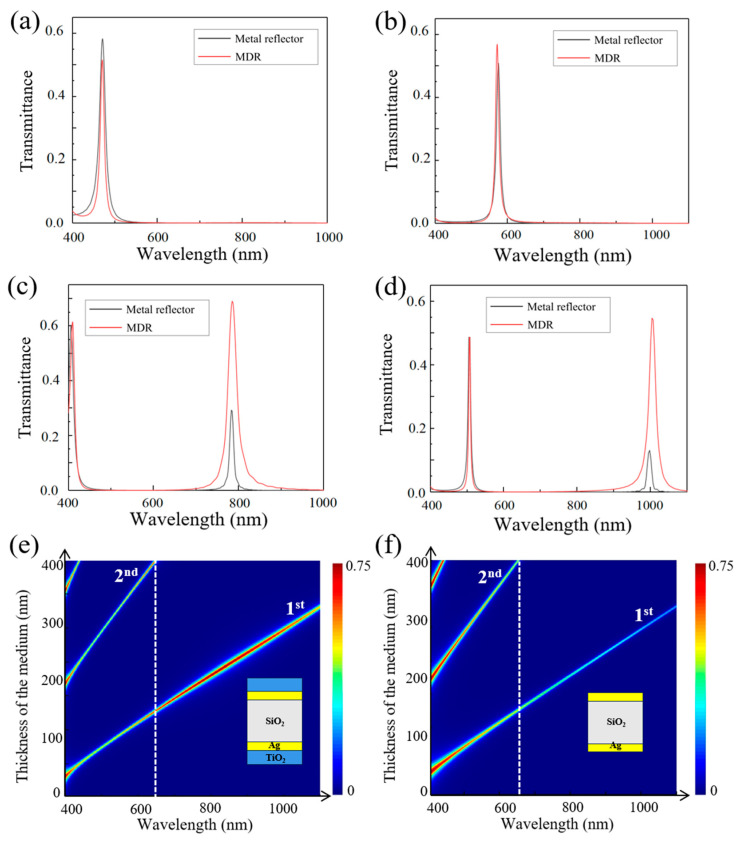
A comparison of the transmission characteristics over the 400–1100 nm wavelength range between the MDR-constructed resonator and a metal-only resonator with the same thickness and cavity length, which work at (**a**) 450 nm, (**b**) 580 nm, (**c**) 780 nm, and (**d**) 1000 nm. (**e**) Transmittance of the MDR-constructed resonator of different medium thicknesses and (**f**) the metal-only resonator. The white dashed line at 650 nm indicates the boundary between the multiple-resonance-peaks band and the single-resonance-peak band.

We employed an MDR composed of 76 nm TiO_2_ and 40 nm Ag to construct a resonator, and its performance across the 400–1100 nm range was validated through simulations. Four cavity lengths were selected to compare the MDR-constructed resonator and the metal-only resonator. As illustrated in Figure 3, the MDR-constructed resonator shows a slight reduction in transmittance within the 400–500 nm range, an unavoidable trade-off to achieve stable, high-transmission coverage across a broader spectrum. This trend is consistent with the transmittance behavior shown in Figure 2a, which exhibits a drop in this spectral region. However, in the 500–1100 nm range, the MDR-constructed resonator exhibits a superior performance. For a transmission peak centered at 580 nm, the MDR-constructed resonator achieves a higher transmittance than the metal-only resonator, with both maintaining the same FWHM. Furthermore, transmission values for the main resonance peaks across the 400–1100 nm range were calculated. As shown in Figure 3e,f, while the transmission peaks of the metal-only resonator sharply decrease at longer wavelengths, the MDR-constructed resonator still has a peak transmittance above 50% in the 650–1100 nm. By selecting the appropriate cavity length, high-transmittance, single-transmission peaks in the 650–1100 nm range can be readily achieved. However, secondary transmission peaks resulting from interference effects may compromise the monochromaticity of the filter. These unintended peaks, occurring within the 400–650 nm range, can induce channel crosstalk and substantially restrict the operational bandwidth of the filters.

### 2.2. Elimination of the Influence of Secondary Transmission Peaks

Due to the inherent constraints of interference principles, the occurrence of secondary peaks in Fabry–Pérot filter structures is unavoidable. Current strategies typically involve either limiting the operational bandwidth to exclude spectral regions with secondary peaks or employing a design to eliminate odd-order resonances. The method of suppressing certain resonances does not fundamentally broaden the FSR. In Fabry–Pérot resonators, the frequency spacing between resonance peaks is fixed and determined by the optical path difference between the reflective surfaces of the cavity, which is influenced by both the refractive index and the thickness of the dielectric material inside the cavity. We propose a novel tuning method that modifies the phase shift experienced by light waves traveling within the resonator. By adjusting the phase difference between waves reflected from the two reflectors, we do not eliminate other resonances but significantly widen the center wavelength spacing between the first and second resonances. When a light wave propagates through a multilayer film system, the reflected light at the interface between two media undergoes an additional phase shift due to reflection [28]:

(6) $$\varphi_{\Delta }=\arctan\left[\frac{i\eta_{0}(CB^{*}-BC^{*})}{\eta_{0}^{2}BB^{*}-CC^{*}}\right]$$

**Figure 4:**
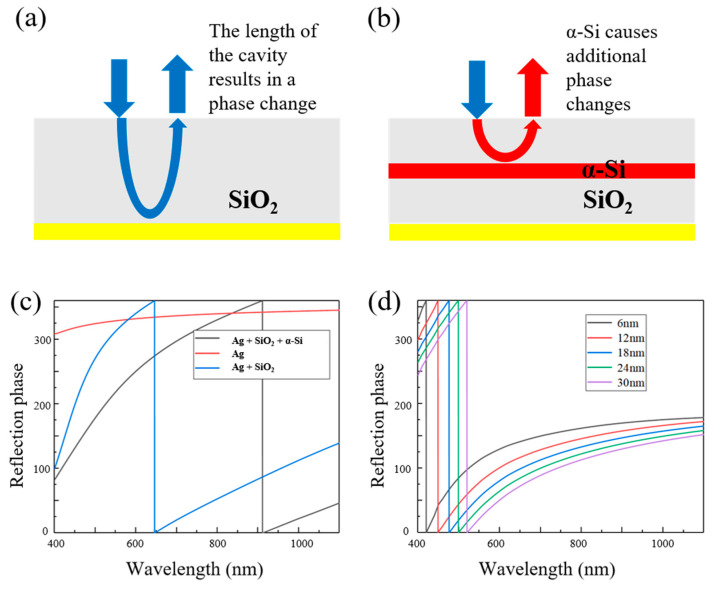
Schematic diagram of reflector phase control: (**a**) Ag + SiO_2_ and (**b**) Ag + SiO_2_ + α-Si; (**c**) the reflection phases for s-polarized light; (**d**) the reflection phases of different α-Si thicknesses for s-polarized light.

By applying this principle, we introduced a high-refractive-index dielectric layer within the resonator to modulate the phase of the light waves undergoing interference in the cavity. This introduces an additional phase increment to the light waves participating in interference within the resonant cavity. Due to the high refractive index of α-Si from the visible to near-infrared, we select α-Si as the high-refractive-index film layer and introduce it into the resonant cavity to control the phase of the reflected light. When the incident light is perpendicular to the surface of the resonant cavity, the reflection phases for s-polarized light are shown in Figure 4c, where it is clear that the structural modification in the cavity causes a shift in the reflection phase. At approximately 400 nm, the reflection phase closely matches that of the Ag + SiO_2_ structure. However, as the wavelength increases, the reflection phase remains consistently lower compared to the Ag + SiO_2_ structure, with the phase difference stabilizing at longer wavelengths. This result demonstrates that during the second reflection, the reflection phase inside the cavity is a cumulative effect, incorporating both the phase modulation of the cavity and the phase contribution from the metal mirror. This behavior directly influences the resonance wavelengths of the Fabry–Pérot cavity. As a result, the resonance wavelength initially expected at 650 nm shifts to 912 nm, while the resonance at 400 nm remains largely unchanged. By introducing a high-refractive-index film for phase modulation, the FSR is significantly increased, greatly expanding the operational bandwidth of the resonator.

As shown in Figure 4d, we also calculated the impact of various α-Si thicknesses on the phase modulation within the resonator. The results indicate that a thicker α-Si layer exhibits stronger phase modulation. However, as the wavelength increases, the modulation effect diminishes. At 400 nm, the phase difference between the thinnest and thickest layers is approximately 2π/5, whereas this difference decreases to around 2π/9 at 1100 nm. Due to the inherent absorption of α-Si, excessively thick layers would lead to a decrease in transmittance. Therefore, to ensure effective phase modulation across 400–1100 nm, the optimal α-Si thickness was determined to be 20 nm.

**Figure 5:**
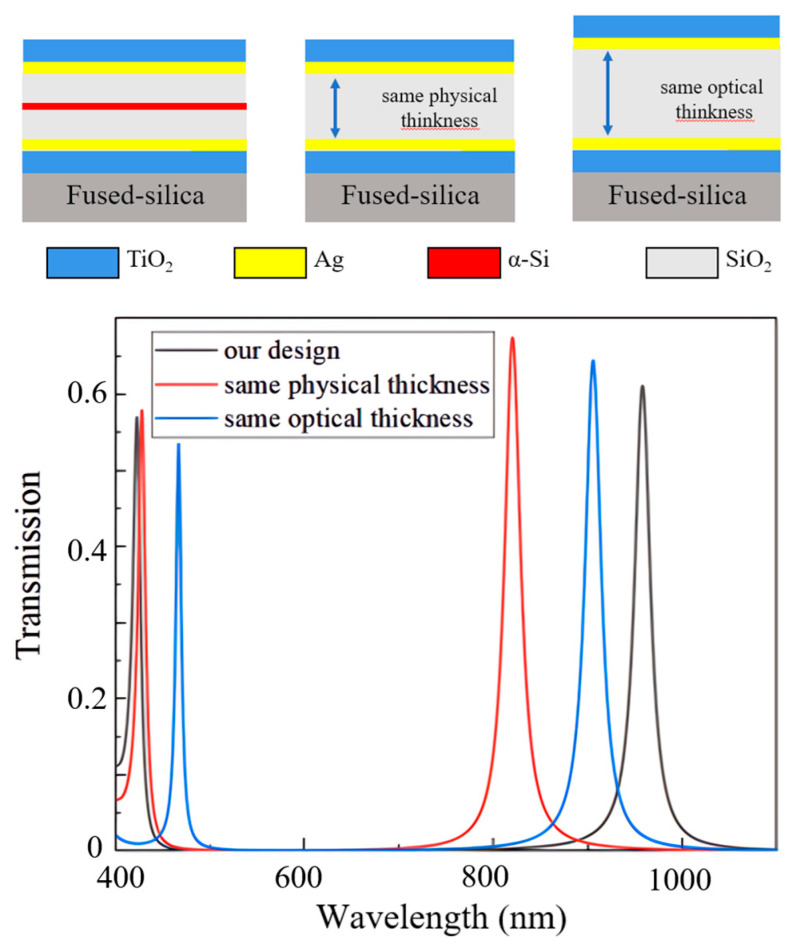
The phase modulation broadens the FSR significantly compared to a conventional Fabry–Pérot filter of the same physical and optical thickness.

To further demonstrate the bandwidth expansion resulting from the phase modulation, we simulated the transmission spectrum of the proposed filter structures, as shown in Figure 5. We also compared the transmission spectra of a single-medium resonator with the same physical thickness and a single-medium resonator with the same optical thickness. The results show that the spacing between adjacent resonance wavelengths in the phase-modulated filter structure is increased by at least 200 nm, representing an expansion of over 30%. This phase modulation strategy significantly increases the FSR and substantially broadens the operational bandwidth of the Fabry–Pérot filter.

### 2.3. Design of Multi-Channel Filtering Structure

**Figure 6:**
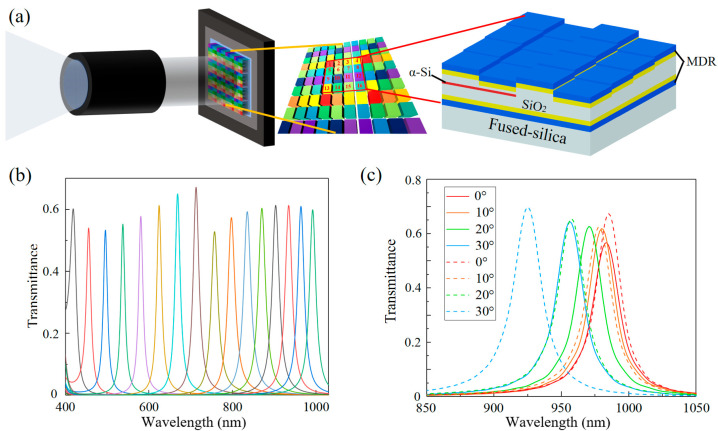
(**a**) Schematic diagram of the filter structure of the array; (**b**) transmission spectra of 16 channels; (**c**) the response of the structure to the angle of incidence. The solid line is the resonant cavity with the α-Si layer introduced, and the dotted line is the resonant cavity without the α-Si layer.

The dielectric–metal resonant cavity we developed significantly enhances the transmittance of metal-based Fabry–Pérot filters within the 400–1100 nm range. By integrating phase modulation techniques, we successfully extended the FSR of the resonator. This dual approach enables the design of an MSFA that offers narrow bandwidth, high transmittance, and strong monochromaticity across the 400–1100 nm range. For an MDR using a single dielectric material, resonance modes are only maintained within the 400–700 nm range. To extend the resonance beyond 700 nm, phase modulation is essential. As illustrated in Figure 6, we designed a structure with the bottom three layers consisting of 76 nm TiO_2_, 40 nm Ag, and 90 nm SiO_2_. On this foundation, additional SiO_2_ layers with varying thicknesses were added to adjust the central wavelength of the primary transmission peak, covering the 400–700 nm range. For the 700–1100 nm range, we introduced a 20 nm α-Si layer above the base three-layer structure, followed by multiple SiO_2_ layers of varying thicknesses to fine-tune the position of the primary peak. The transmission spectra for 16 (4 × 4) different central wavelengths are shown in Figure 6b. The results confirm that the MSFA achieves over 50% transmittance across the entire working bandwidth, with FWHM values consistently below 25 nm. Additionally, we calculated the response of the structure to varying incident angles, as shown in Figure 6c. The results indicate that the resonant cavity with the α-Si layer shows a smaller shift in central wavelength with a changing incident angle. At an incident angle of 30°, the α-Si layer structure exhibits a central wavelength shift of less than 30 nm, significantly smaller than that of the structure without the α-Si layer (60 nm). This is because the resulting phase difference is not solely due to the optical path difference; with changes in the incident angle, the additional optical path difference is minimized, leading to a reduced central wavelength shift.

## 3. Results and Discussion

**Figure 7:**
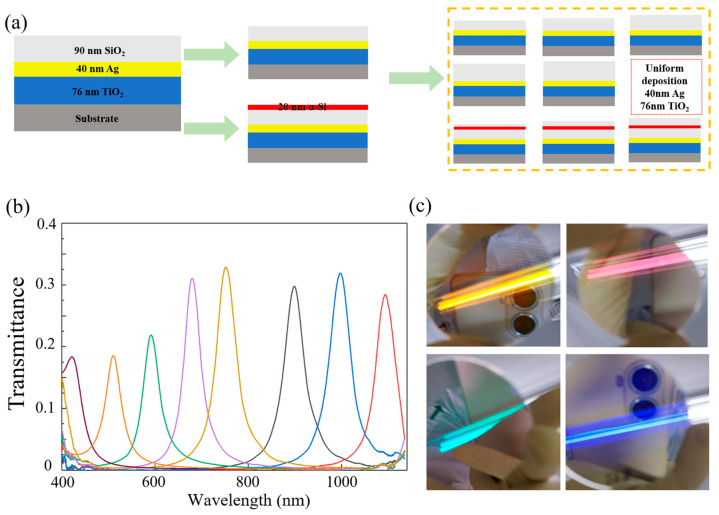
(**a**) Schematic diagram of the filter verification process. (**b**) The measured transmittance of each filter. (**c**) Some of the actual filters.

Given the extensive research and fabrication efforts on metal-based Fabry–Pérot filters by numerous researchers, to optimize the fabrication process and costs, we simplified our validation experiment by using eight filter sets to verify our proposed MSFA structure. The structure is composed of seven layers: 76 nm of TiO_2_, 40 nm of Ag, 90 nm of SiO_2_, 20 nm of the α-Si layer, SiO_2_ of thickness *h*, 40 nm of Ag, and 76 nm of TiO_2_. Since only the 20 nm α-Si layer and SiO_2_ with thickness *h* are varied, the fabrication process only requires adjustments to these two layers for each filter. To closely align with MSFA-manufacturing processes, all other layers were deposited in a single batch. We used thermal evaporation to deposit 76 nm TiO_2_, 40 nm Ag, and 90 nm SiO_2_ on eight substrates. Three of these were then coated with a 20 nm α-Si layer using PECVD. The remaining five filters were divided into five groups, with each group receiving a SiO_2_ layer of 0, 30, 60, 90, or 120 nm. The three filters with the 20 nm α-Si layer were placed into the second, third, and fourth groups for simultaneous deposition of 30, 60, and 90 nm of the SiO_2_ film. Finally, all eight filters underwent the final deposition of 40 nm of Ag and 76 nm of TiO_2_. This process substantially reduces the complexity of large-scale manufacturing. The resulting filters are shown in Figure 7a.

**Figure 8:**
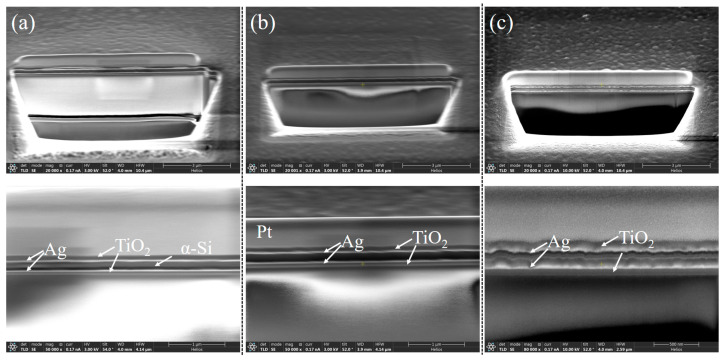
The cross-sectional SEM (FEI, Helios G4 CX) images of the filter based on MDR and phase control by the high refractive index α-Si layer inside the cavity: (**a**) shows the filter structure with a 20 nm α-Si film inside the cavity, while (**b**,**c**) show the structure only with SiO_2_ film.

We evaluated the transmission spectra of eight filters using a spectrometer (IdeaOptics, FX2000-RD, spectral range: 350–1100 nm, resolution: 1.4 nm@546 nm), as depicted in Figure 7b. Across the 400–1100 nm wavelength range, all filters exhibited single-peak transmission. The transmission and FWHM across the operational bandwidth demonstrated a relatively stable performance. However, as shown in Table 1, a comparison with the simulation data revealed a notable decrease in transmission and an increase in the FWHM in the experimental results. This discrepancy is attributed to the higher surface roughness of the thin films, which is clearly represented in Figure 8c [35,36,37]. In addition, all central wavelengths exhibited varying degrees of redshift, primarily due to the actual thickness of SiO_2_ and α-Si being greater than the designed thickness. Process constraints during fabrication impart a degree of surface roughness to the mirrors, which in turn causes slight variations in the cavity length. Consequently, the cavity length deviates slightly from the ideal uniform thickness, following a normal distribution around the ideal value. These variations create multiple resonance modes at different central wavelengths within the cavity, resulting in reduced transmittance and a broader transmission peak [38]. It is important to note that this broadening is not a design flaw but rather a result of the distribution of transmitted light energy over a wider range around the central wavelength. To quantify this effect, we integrated the transmission spectra of the first five filters, yielding energy values of 1.89, 2.16, 2.5, 2.97, and 3.84, respectively. For the corresponding simulation results with the same central wavelengths, we obtained values of 2.05, 2.16, 2.5, 2.73, and 2.92. The close agreement between these datasets suggests that the observed reduction in the filter performance is primarily due to the surface roughness of the films introduced during fabrication. Previous studies, such as those by Jingyuan Zhu et al., have successfully addressed similar issues, showing good agreement between simulated and actual results [33]. We are optimistic that further optimization of the fabrication process will enhance the experimental results for our proposed design. We are actively pursuing collaborative opportunities to overcome these fabrication challenges and achieve improved performance.

**Figure 9:**
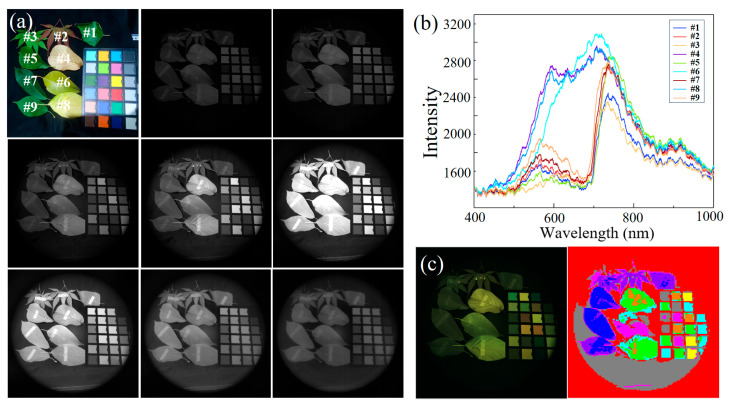
(**a**) RGB plot and grayscale plot of eight filters from the actual test scene. (**b**) Reflectance spectra of each leaf in the scene from the FX2000-RD spectrometer. (**c**) Grayscale image restoration to a color image and K-means classification results for the whole scene.

In applications across the visible to near-infrared spectrum, a common case is distinguishing genuine plants from counterfeit ones using the red-edge effect. We mounted the filters onto a CMOS detector (Gpixel, GSENSE400BSI, pixels: 2048 × 2048) and imaged a scene illuminated by a halogen lamp, which included a black background cloth, a standard color chart, and nine real leaf samples. The grayscale images clearly reveal the double-sided tape affixed to the backs of the leaves, especially noticeable on Leaf 6, as shown in Figure 9a. In certain channels, the tape is not visible on the back of Leaf 6, but in the infrared grayscale images, it is prominently observed. The visibility discrepancy arises because the leaf reflects a large portion of visible light. In contrast, infrared light transmits through the leaf and is then strongly reflected by the tape on the back.

We constructed a feature data cube from grayscale images captured using different filters and utilized this data cube for basic target classification within the scene. To demonstrate the advantages of the ultra-wide bandwidth device, we employed the simplest unsupervised K-means clustering classification method. This classification model features a simple computational process and an extremely fast response speed. Figure 9b shows the measured reflectance spectra of nine leaves in the scene. Figure 9c presents the K-means classification results. Due to significant variation in the eight feature values for the same target leaf within the scene, the classification of the fourth, sixth, and eighth groups of fallen leaves was inaccurate, as these groups could not be clearly distinguished from certain areas of the color chart. However, in the fresh leaf samples, the feature values were characterized by the distinctive reflectance surge of plants at 750 nm. Consequently, the classification results show that the purple and blue–purple regions representing fresh leaves did not appear in other areas in the figure. Nevertheless, due to the limited number of features and classes, the classification of the color blocks on the chart was approximate, and there remains a lack of detailed distinction between different plants and the color chart.

**Figure 10:**
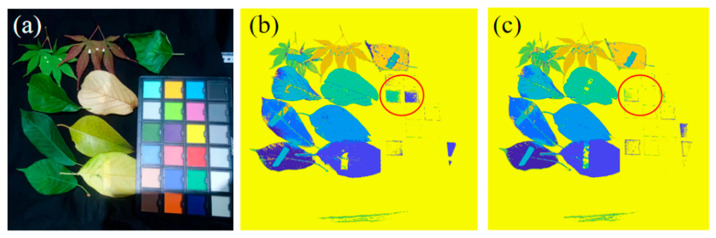
(**a**) Image of the test scene. (**b**) Classification results using only the visible band feature information for training. (**c**) Classification results using feature information from the 400–1100 nm band for training.

To further highlight the advantages of multispectral imaging from the visible to near-infrared, we processed the multispectral image data cube by extracting grayscale information from filters 1 through 5 to create a visible-band multispectral image. We selected 1000 pixels for labeling, constructing a training dataset. Similarly, we constructed a training dataset for the combined visible and near-infrared feature values. We implemented a basic multi-layer perceptron network with two fully connected layers. The network received pixel feature data as input and produced classification labels as output. Both datasets were used to iteratively train the model, which was then applied to classify the multispectral data. The classification results, shown in Figure 10, demonstrate that both the visible and the visible-to-near-infrared imaging schemes can accurately classify leaf types and their condition. Notably, in the area circled in red, the gray and light green regions of the color chart were misclassified as leaves in the visible imaging scheme, while the visible to near-infrared scheme correctly identified them as non-leaf targets. This indicates that increasing the working bandwidth of the imaging device, even when using the same algorithm, enhances the ability of the system to differentiate between visually similar targets. This performance improvement has broad applications in fields such as live target detection and camouflage recognition.

The actual spectral resolution and efficiency measured are lower than the theoretical values, primarily due to limitations imposed by the deposition process [39]. These thin-film defects could be mitigated with the use of advanced equipment and refined CMOS fabrication techniques. Despite these challenges, our results demonstrate that the eight spectral channels cover an extended visible bandwidth, with each integrated filter exhibiting a single-channel response. While reducing the thickness of the metal layer can achieve higher peak transmittance and minimize channel broadening caused by surface roughness, it may also lead to an increase in FWHM, potentially degrading the device performance.

Nevertheless, our results show significant improvements in the operational bandwidth and energy efficiency compared to conventional metal and dielectric Fabry–Pérot filters. Although we have only presented a validation scheme for the filters, the existing research suggests that grayscale etching or multi-step etching could be employed to fabricate array-based single-chip filter structures. The high-precision Electron Beam Lithography (EBL) 3D grayscale technique shows great potential for providing additional spectral channels. In industrial production, grayscale masks for multi-channel filters can also be produced using EBL, enhancing both the efficiency and stability of filter manufacturing [40].

## 4. Conclusions

In this study, we theoretically proposed and experimentally validated a broadband multispectral filter structure based on an MDR Fabry–Pérot resonator. We introduced a phase control mechanism to extend the operational bandwidth from 400 nm to 1100 nm, encompassing a broader range of target information. Compared to traditional Fabry–Pérot multispectral filter arrays, our design offers an ultra-wide operational bandwidth, overcoming the trade-off between FSR and FWHM. Our developed filter does not rely on angle-sensitive diffraction structures, making it relatively insensitive to angle variations up to 30°. Although we have only validated the 8-channel design through preliminary testing and have not yet fabricated the 16 (4 × 4) MSFAs, the results successfully demonstrate the effectiveness of our design principle. Current challenges, such as the surface roughness of films caused by fabrication limitations, have led to a broader FWHM, lower peak transmittance, and insufficient performance in some bands compared to theoretical predictions. Addressing these issues is a key goal for future work, alongside high- and low-temperature and aging testing to ensure industrial applicability.

This filter array also features high customizability and is compatible with current micro-pixel dimensions. Its practical manufacturability is high, requiring only a basic deposition technique for MSFA fabrication. Building on the existing Fabry–Pérot MSFA-manufacturing processes, this structure can be produced in large quantities at a low cost. Furthermore, the phase control mechanism within the cavity can be extended to other fields to enhance spectral detection capabilities, such as gradient filters. In multispectral snapshot imaging applications, this design can directly replace traditional Bayer color filters, enabling single-chip integrated multispectral CMOS sensors with a wide operational wavelength range and high imaging contrast and quality without relying on complex algorithms. The design principles demonstrated in this study can be easily adapted to other wavelength ranges, such as near-infrared and infrared. At longer wavelengths, this approach could provide an even broader wavelength range and be easier to manufacture. Thus, our device presents a complete system for snapshot spectral imaging, achieving a high optical performance over an extensive bandwidth. It also provides a low-cost, compact, and portable solution for various applications, from camouflage detection imaging to remote sensing.

## Figures and Tables

**Table 1:** Comparison between the simulation data and experiment data of eight filters.

Data Source	Data Type	#1	#2	#3	#4	#5	#6	#7	#8
Simulation	Central Wavelength (nm)	415	490	571	655	713	784	888	982
	Peak transmission (%)	59	53	57	63	64	55	59	58
	FWHM (nm)	20	14	14	18	19	22	24	22
Experiment	Central Wavelength (nm)	421	509	590	676	755	898	988	1094
	Peak transmission (%)	18	18	22	31	33	28	31	28
	FWHM (nm)	47	41	40	40	46	45	45	50

## Data Availability

Data are contained within the article.
